# Aerogels Prepared from Enzymatically Modified *Canna edulis* Starch: Structure and Cyanidin-3-glucoside Adsorption Performance

**DOI:** 10.3390/gels12090753

**Published:** 2026-08-22

**Authors:** Xiangjie Zhao, Xinrui Huang, Jin Yang, Cancan Shao, Yang Li, Rongling Yang

**Affiliations:** School of Life Science and Food Engineering, Huai’an University, Huai’an 223003, China; zhaoxiangjie@hau.edu.cn (X.Z.);

**Keywords:** canna starch, aerogel, enzymatic modification, adsorption, cyanidin-3-glucoside

## Abstract

Although canna (*Canna edulis* Ker) starch possesses high swelling power, it remains an underutilized resource. This study aimed to engineer highly porous, food-grade canna starch aerogels as carriers for cyanidin-3-glucoside (C3G), incorporating enzymatically modified starch fractions prepared via α-amylase hydrolysis followed by lyophilization. Moderate enzymatic modification (sample AG1) effectively tailored the aerogel architecture by selectively removing amorphous regions. This targeted hydrolysis yielded a highly interconnected, hierarchical porous network with a high porosity (92%). Importantly, although this architectural transformation increased the average macroscopic pore size, it preserved mechanical integrity, as evidenced by a compressive strength exceeding 4000 kPa. In contrast, excessive hydrolysis led to pore collapse and structural failure. The AG1 aerogel exhibited a markedly enhanced C3G equilibrium adsorption capacity (43.9 mg/g), outperforming the native starch aerogel (36.0 mg/g). Adsorption data adhered to pseudo-second-order kinetics, indicating that this model provided a better description of the adsorption process, while the underlying adsorption mechanism may involve interactions between C3G and the aerogel matrix, potentially including hydrogen bonding. These results suggest that controlled enzymatically hydrolysis may provide a useful strategy for modulating the hierarchical microstructure of canna starch aerogels, thereby supporting their potential as effective carriers for sensitive bioactive compounds in functional food systems.

## 1. Introduction

Aerogels are a class of three-dimensional porous materials characterized by interconnected pore networks. They exhibit unique properties, including high porosity, large specific surface area, and low density, making them promising materials for various applications [1]. Owing to these characteristics, aerogels have been widely applied in fields such as electronics, optics, catalysis, and thermal insulation [2]. Early research on aerogels was primarily based on inorganic compounds [3]. However, in recent years, the increasing global demand for biodegradable materials has made the utilization of natural polysaccharides for aerogel preparation an increasingly attractive option [4]. As a natural polysaccharide, starch possesses several advantageous characteristics, including abundant availability, low cost, biodegradability, and excellent biocompatibility. These attributes render starch a promising candidate for the preparation of aerogels [5].

Starches from different botanical sources exhibit variations in chain structure, amylose/amylopectin ratio, and functional group composition [6]. These variations can significantly influence the structural characteristics and physicochemical properties of starch-based aerogels, particularly after enzymatic modification, which can alter the branch chain distribution, crystallinity, and rheological behavior of starch matrices [7]. Starch-based aerogels with varying amylose contents (ranging from 0% to 100%) have been reported to exhibit significant differences in morphology, density, surface area, and thermal conductivity [8]. This indicates that the concentration of amylose plays a key role in determining both the structure of the aerogel and its insulation performance. The food industry has a considerable demand for starch. In certain instances, unconventional starches can partially or fully replace traditional starches [9]. Consequently, the food industry needs to identify new sources of starch.

*Canna edulis* Ker is a member of the *Cannaceae Juss.* family and the *Canna L.* genus, originally cultivated in the Andes region of South America. Over time, it has been introduced to various countries across Asia and Africa. This species serves as a non-traditional source of starch and is extensively cultivated in numerous provinces of China, particularly in Yunnan [10]. The tuber of canna (*Canna edulis* Ker) is rich in starch, making it a valuable starch crop with considerable economic value. As a highly adaptable starch source, canna has considerable potential to replace traditional food raw materials, thereby promoting the agricultural economy in mountainous regions and expanding its applications across various industrial sectors [11].

Currently, aerogels prepared with natural starch exhibit insufficient porosity and non-uniform pore structures [12], which significantly limits their potential applications. However, moderate structural modifications to the starch framework through enzymatic treatment can enhance both its porous architecture and adsorption capabilities. Dura et al. [13] observed by scanning electron microscopy (SEM) that α-amylase or amyloglucosidase treatment induced porous structures in starch granules, with obvious surface cavities and erosion compared with untreated starch. Research has demonstrated that α-amylase hydrolysis of tapioca starch before aerogel formation reduced amylose content by 9.46% and increased the adsorption capacity of starch aerogels by 25.40% compared with untreated samples, owing to the regulation of starch molecular structure [14]. Similarly, Jiang et al. [15] demonstrated that microwave-assisted enzymatic hydrolysis improved the absorption properties of porous starch, with water and oil absorption capacities increasing from 110.99% and 133.11% to 128.29% and 143.30%, respectively. Enzymatic modification broadens the application scope of starch-based aerogels, thereby providing a foundation for their use in food packaging and preservation materials [16]. In addition to structural optimization, starch-based aerogels have shown considerable potential as carriers for bioactive compounds due to their abundant hydroxyl groups and tunable pore networks.

Anthocyanins are excellent natural antioxidants and functional active ingredients, with reported benefits related to antioxidative and anti-inflammatory activities and cardiovascular health [17]. However, anthocyanins are highly sensitive to environmental factors, making the enhancement of their stability a critical challenge in functional food applications. Among anthocyanins, cyanidin 3-glucoside (C3G) is the most widely distributed [18]. In this context, assessing the adsorption behavior of C3G onto both native and α-amylase-modified canna starch aerogels provides essential insights into their functional performance and broadens the applicability of canna-derived materials in value-added applications.

**Scheme 1:**
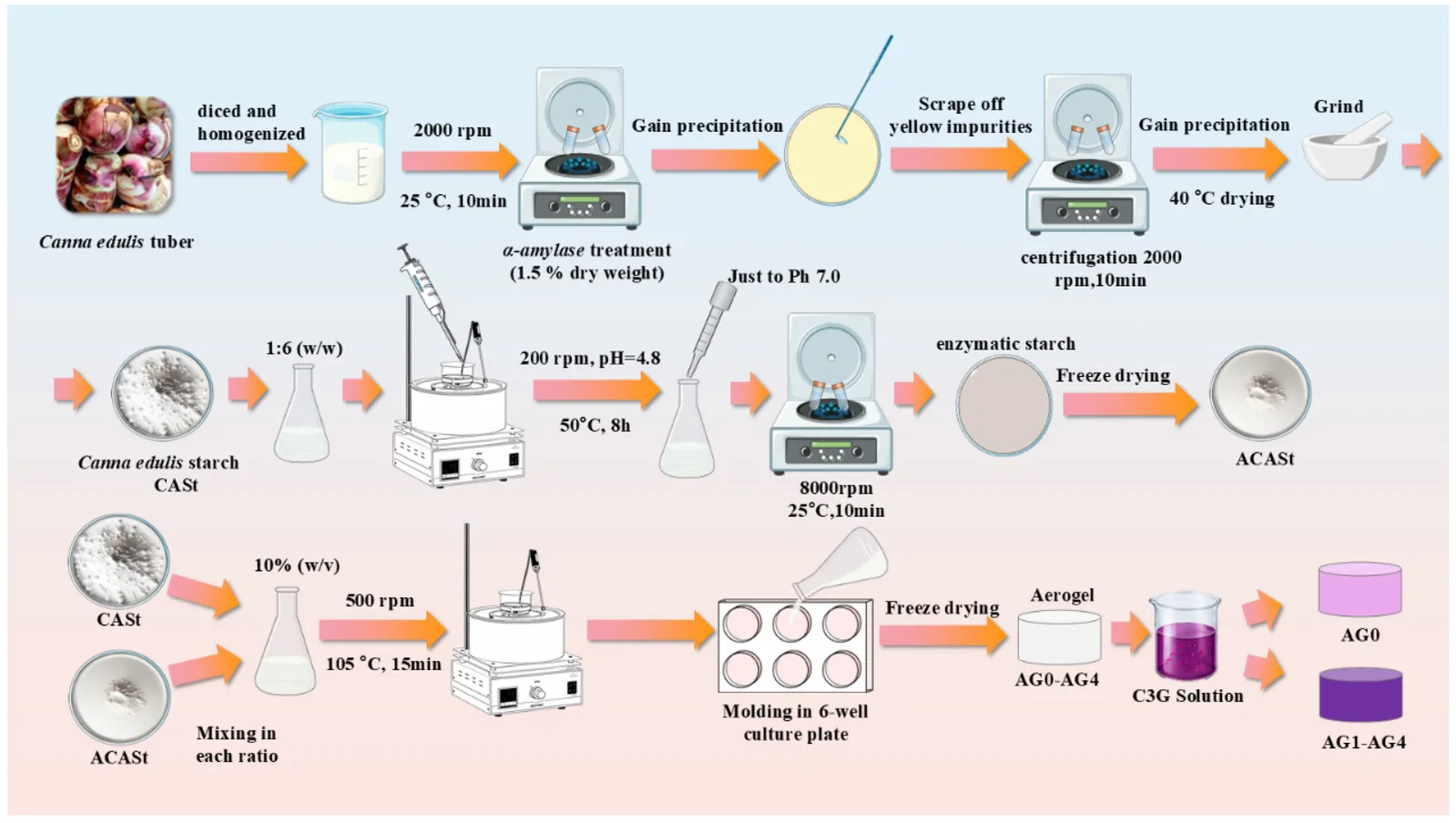
Schematic diagram of the preparation of canna starch-based aerogels.

In summary, as shown in Scheme 1, this study focuses on canna starch. The aim is to address the problems of low porosity and inhomogeneous pore structure in native starch aerogels by controlled α-amylase hydrolysis, and to explore its potential for preparing high-performance aerogels. The physicochemical properties of native canna starch were systematically characterized first. Subsequently, enzymatic modification was performed to construct a highly interconnected porous structure, and the structural, morphological, thermal, and mechanical properties of unmodified and modified canna starch aerogels were compared and analyzed. The results provide insights into how enzymatic hydrolysis regulates the porous structure, providing theoretical support for the development of canna starch-based porous adsorbents and the high-value utilization of this characteristic agricultural product.

## 2. Results and Discussion

### 2.1. Physicochemical and Morphological Characterization of Canna Starch

The extraction yield of canna starch on a dry basis ranged from 57% to 62%, which is slightly lower than the typical yields (65–80%) reported for commercial corn and wheat starches. This variance primarily reflects the anatomical characteristics of *C. edulis* storage rhizomes. Unlike cereal endosperms, *C. edulis* starch granules are embedded within a complex matrix enriched with mucilage, pectic substances, and coarse dietary fibers. This composition creates stronger associations between the granules and cell walls, making complete granule liberation during extraction more difficult. In contrast, corn and wheat feature more loosely organized starch–protein matrices and highly optimized extraction protocols. A comparative analysis of macromolecular constituents (Table 1) revealed distinct characteristics. Canna starch exhibited a higher moisture content than the commercial starches, suggesting differences in hygroscopic behavior. The ash content was markedly lower than in wheat starch but notably higher than in corn starch. Notably, the crude protein content of canna starch was significantly higher than both reference starches, likely resulting from intrinsic differences in the protein matrix or incomplete removal. Conversely, its crude fat content was significantly lower, suggesting an inherently reduced capacity for lipid complexation. Regarding polysaccharide composition, canna starch exhibited a significantly lower amylose content, and a correspondingly higher amylopectin content, compared to wheat and corn starches. Despite these structural differences, the total starch content of canna starch remained exceptionally high and comparable to the commercial references [19]. Collectively, these compositional distinctions position canna starch as a promising precursor for aerogel applications. In particular, it showed markedly lower amylose and higher amylopectin contents, together with moderate protein and minimal lipid levels.

The dominant amylopectin fraction facilitates the formation of an extensive polymeric network that supports a robust hydrogel matrix, enhancing swelling capacity and mechanical integrity [20]. Furthermore, the lower amylose content reduces retrogradation tendencies that could compromise long-term gel stability, while the minimal lipid content promotes unhindered polymer interactions during gelation and drying, which are essential for aerogel microstructure development [21].

**Figure 1:**
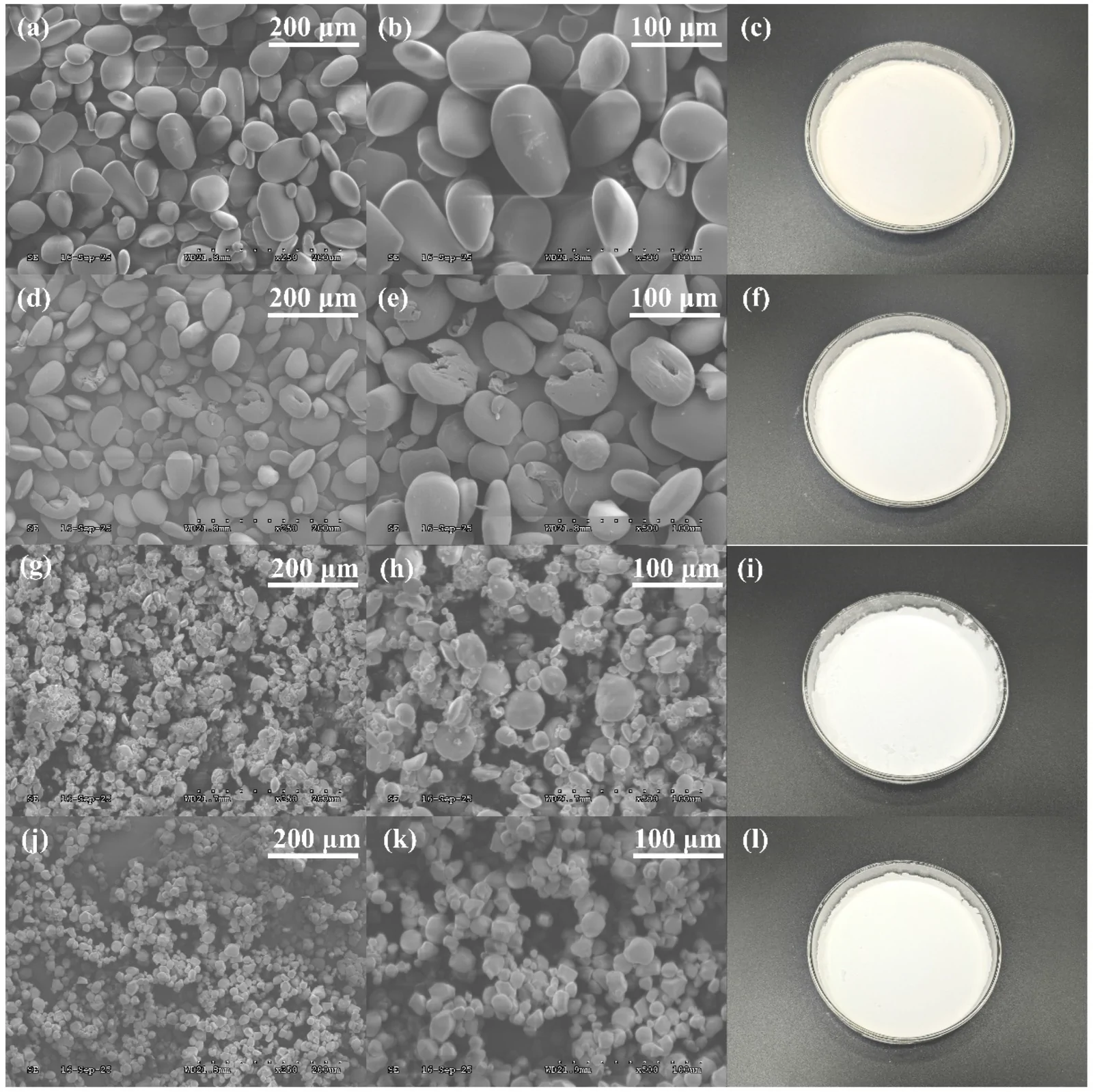
Micrographs and appearance characteristics of canna starch (**a**–**c**), enzymatically modified canna starch (**d**–**f**), wheat starch (**g**–**i**), and corn starch (**j**–**l**) samples.

Scanning electron micrographs (Figure 1) illustrate the morphological differences among the starches. Native canna starch granules exhibited smooth surfaces with oval-to-elliptical shapes and a particle size ranging from 10 to 20 μm, notably larger than corn (5–8 μm) and wheat (4–6 μm) starches. This larger size confers a lower surface-to-volume ratio, which influences gelatinization behavior and facilitates more pronounced structural modifications during the development of porous features [14,22,23]. Following enzymatic treatment, the ACASt granules exhibited distinctive surface erosion, characterized by pores and cavities distributed throughout their exterior. This morphology may result from the preferential hydrolysis of amorphous regions by α-amylase, leaving the crystalline lamellae relatively intact. Such modifications increase both specific surface area and porosity [13]. Consequently, ACASt granules act as effective nucleation sites and templates that facilitate the assembly of porous aerogel frameworks, ultimately enhancing internal void volume and gas adsorption potential. In addition to enzymatic modification, previous studies [24] have shown that structural reinforcement strategies, such as nanocellulose incorporation, can further improve the integrity and morphological stability of starch aerogels, particularly by preventing pore collapse during drying.

### 2.2. Hydration Properties of Native Canna Starch

**Figure 2:**
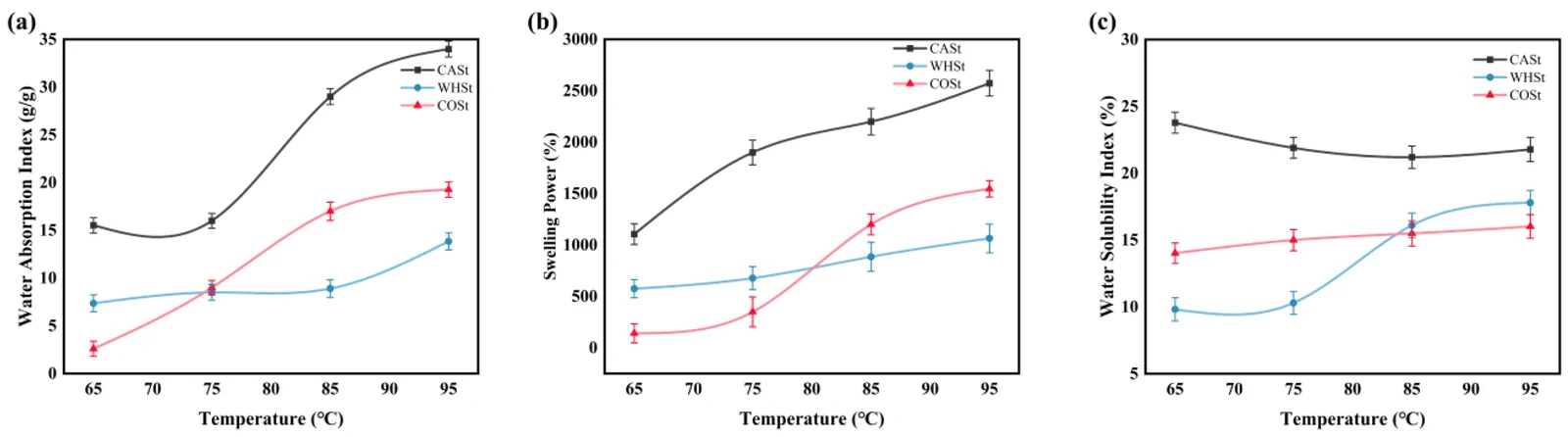
Swelling power (**a**), water absorption index (**b**), and water solubility index (**c**) of native canna starch (CASt), wheat starch (WHSt), and corn starch (COSt) at temperatures of 65 °C, 75 °C, 85 °C, and 95 °C.

In Figure 2, the swelling power (SP), water absorption index (WAI), and water solubility index (WSI) of native canna, wheat, and corn starch samples are presented at temperatures of 65, 75, 85, and 95 °C. The SP, WAI, and WSI of native canna starch were evaluated over this temperature range and compared with those of wheat and corn starches. The results indicated that native canna starch exhibited significantly higher SP values than both control starches at all tested temperatures, increasing from approximately 1100% at 65 °C to around 2600% at 95 °C. This pronounced swelling capacity may indicate that native canna starch granules possess a relatively open granular architecture along with weaker internal associations between amylose and amylopectin, which facilitate rapid water penetration and granule expansion. In contrast, wheat starch displayed moderate SP values ranging from 550% to 1050%, showing a gradual increase dependent on temperature. Corn starch exhibited very low SP at 65 °C (~150%) but demonstrated a sharp rise after reaching temperatures above 75 °C, ultimately achieving 1550% by the time it reached 95 °C. This behavior is consistent with its higher gelatinization temperature and the presence of more ordered crystalline regions.

A similar trend was observed for WAI. Native canna starch consistently showed the highest water-binding capacity, starting at approximately 15–16 g/g. After a slight dip at around 70 °C, WAI rose steeply above 75 °C, peaking at 34 g/g. The strong correlation between SP and WAI in native canna starch underscores the role of granule swelling as the primary driver for water uptake during gelatinization. Wheat starch exhibited a steady increase in WAI from 2.5 g/g to 19.5 g/g, while corn starch rose modestly from 7 g/g to 14 g/g, notably displaying a minor plateau between 75 °C and 85 °C. For WSI, native canna starch showed the highest solubility among the three starches at lower temperatures. Solubility then declines slightly to approximately 21% at 85 °C before stabilizing. This implies that, despite extensive swelling properties, leaching of soluble fractions such as amylose and small amylopectin chains was somewhat controlled. In comparison, wheat starch displayed a gradual increase in WSI from 14% to 16% across the temperature range examined. Corn starch maintained low solubility levels until reaching about 75 °C before sharply increasing to 18% at 95 °C; this rise may be associated with the gelatinization-induced release of amylose.

The starch hydration properties indicate that native canna starch possesses an exceptional combination of high swelling capacity, strong water-binding ability, and relatively stable solubility characteristics that distinguish it from conventional cereal starches. Such properties are advantageous for applications requiring rapid hydration along with high viscosity and thickening efficiency; however, the relatively high baseline solubility may influence retrogradation behavior [25], considerations that should be taken into account during the production of hydrogels and aerogels.

### 2.3. Fourier Transform Infrared Spectroscopy (FTIR) Analysis

**Figure 3:**
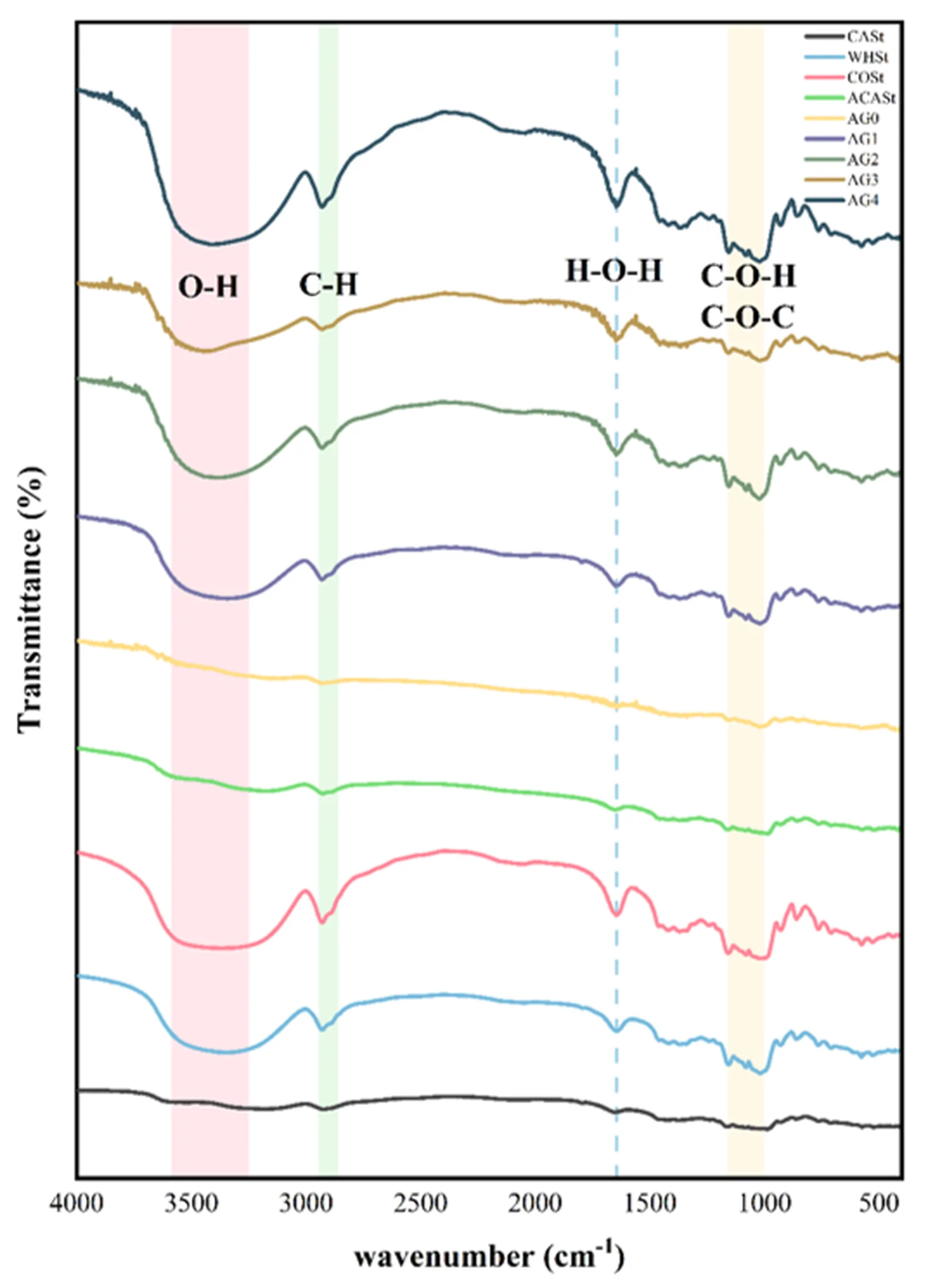
FTIR spectra of starch samples and aerogels AG0—AG4.

FT-IR spectra (Figure 3) revealed that the native starches, enzymatically modified samples, and composite aerogels shared similar absorption profiles without the appearance of new bands. This suggested that enzymatic treatment and gelation did not result in the formation of detectable new functional groups. The observed structural changes were mainly related to variations in intermolecular interactions and molecular organization. The broad -OH stretching band (3200–3600 cm^−1^) was broader in modified and aerogel samples, suggesting changes in hydroxyl group environments that may be associated with variations in intermolecular hydrogen-bonding interactions [26]. Concurrently, shifts in the C-H stretching bands (2926–2930 cm^−1^ and 2880–2896 cm^−1^) [27] indicated possible conformational adjustments of starch chains during enzymatic modification and aerogel formation. The enhanced intensity of the H-O-H bending vibration near 1640 cm^−1^ in the aerogels suggested stronger interactions with absorbed water, which may be related to their porous structure and increased accessibility of water-binding sites [28].

In the fingerprint region (1200–900 cm^−1^), characteristic bands of glycosidic linkages became broader and weaker in the modified and aerogel samples [29], suggesting changes in the local molecular arrangement of starch chains. These spectral changes were consistent with the decreased intensity of peaks at 930–950 cm^−1^ (α-1,4 glycosidic bonds) and at 883 cm^−1^, 821 cm^−1^, and 740 cm^−1^ (pyranose ring and skeletal vibrations) [29].

To assess short-range order semi-quantitatively, the region (800–1200 cm^−1^) was analyzed based on the absorbance values at approximately 995 cm^−1^, 1022 cm^−1^, and 1047 cm^−1^ obtained from the converted spectra, and the absorbance ratios R_1047/1022_ and R_1022/995_ were used as semi-quantitative indicators of short-range structural order and potential changes in the hydrogen-bonding environment, respectively. As shown in Table 2, R_1047/1022_ decreased from 0.9712 (CASt) to 0.8545 (ACASt) after enzymatic hydrolysis, suggesting partial disruption of short-range order [30]. In aerogels, AG0 recovered to 0.9773, while AG1 (1.3718) and AG2 (1.3863) yielded the highest ratios, potentially reflecting selective removal of amorphous regions and enrichment of ordered domains, analogous to lintnerized starch residues. Excessive hydrolysis (AG3, AG4) returned ratios to near-baseline. R_1022/995_ dropped from 1.0120 to 0.7322 (ACASt) and 0.7230 (AG0), potentially reflecting changes in the hydrogen-bonding environment associated with hydroxyl groups during hydrolysis. These ratios give only semi-quantitative insight, and their link to order is non-linear [30]. Overall, the FTIR results suggested that the transformation process induced molecular rearrangement and changes in structural ordering, rather than the formation of new chemical bonds.

### 2.4. Morphology of the Aerogels

The density and porosity of the aerogels are summarized in Table 3. As the ACASt content increased, the density decreased from 0.11 g/cm^3^ in AG0 to about 0.09 g/cm^3^ in AG2-AG4, while the porosity increased from 86% to around 91–92%. This suggests that the overall aerogel structure became more open after ACASt was introduced.

**Figure 4:**
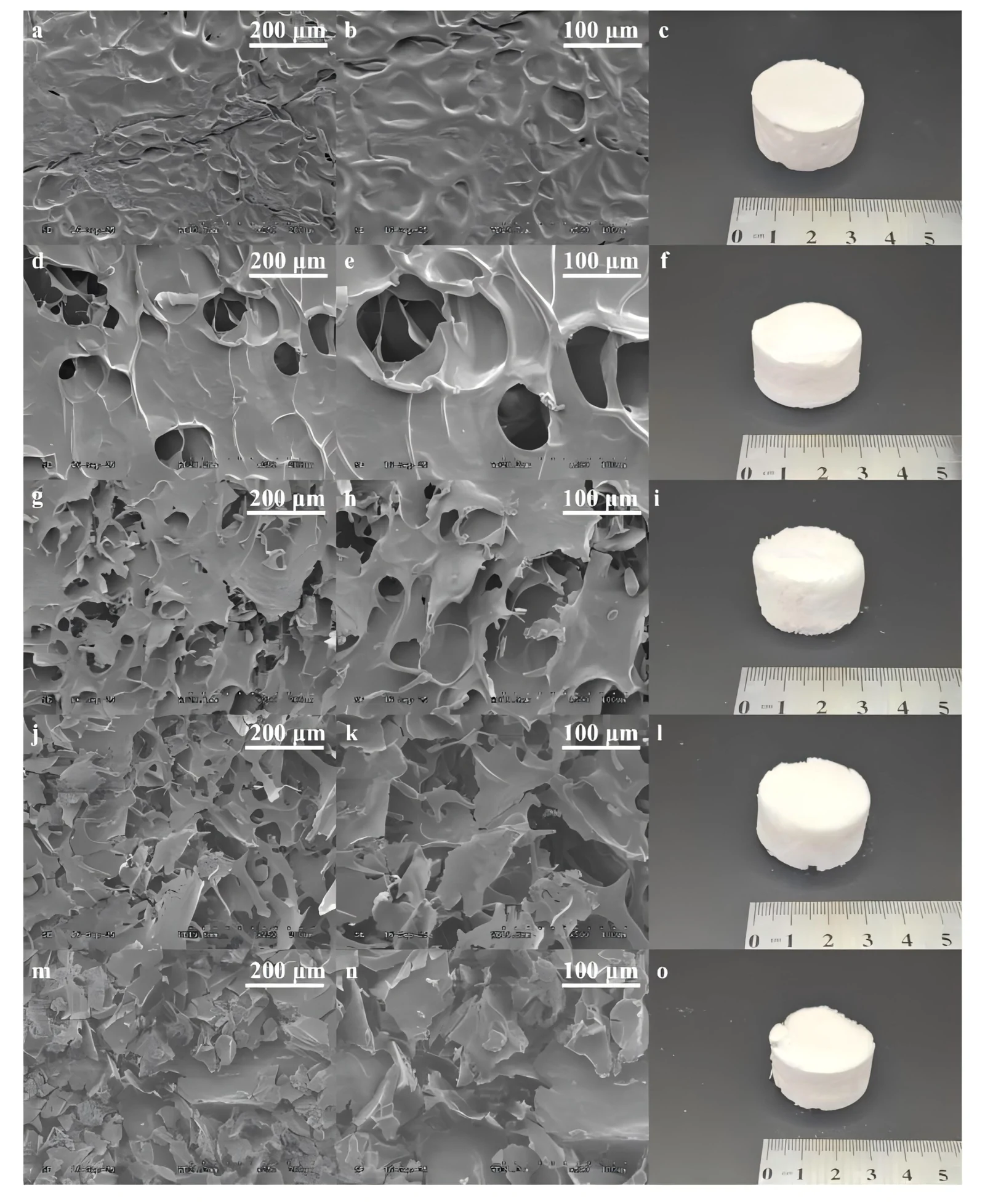
SEM images and macroscopic appearances of canna starch aerogels prepared with different ACASt contents: (**a**–**c**) AG0, (**d**–**f**) AG1, (**g**–**i**) AG2, (**j**–**l**) AG3, and (**m**–**o**) AG4.

SEM images show clear differences among the samples (Figure 4). AG0 had a relatively compact structure, with only a few visible pores, suggesting relatively dense packing of the starch network [31]. After adding ACASt, the structure became more open. In AG1 and AG2, more pores could be seen, and the surface became rougher, but the network was still continuous. This matches the trend in Table 3. When the ACASt content was further increased (AG3 and AG4), the pore structure became less uniform. Some areas showed thinner pore walls and slight collapse. AG4, in particular, presented a more broken and irregular structure, suggesting that too much ACASt may disturb the stability of the gel network during freeze-drying. Even though the porosity values did not change much among AG1-AG4, the SEM results suggest that additional ACASt mainly affected structural uniformity rather than substantially increasing pore formation.

Overall, a moderate amount of ACASt helped form a more open structure, while too much led to some structural disruption.

### 2.5. XRD Analysis of Starches and Enzyme-Modified Canna Starch Aerogels

**Figure 5:**
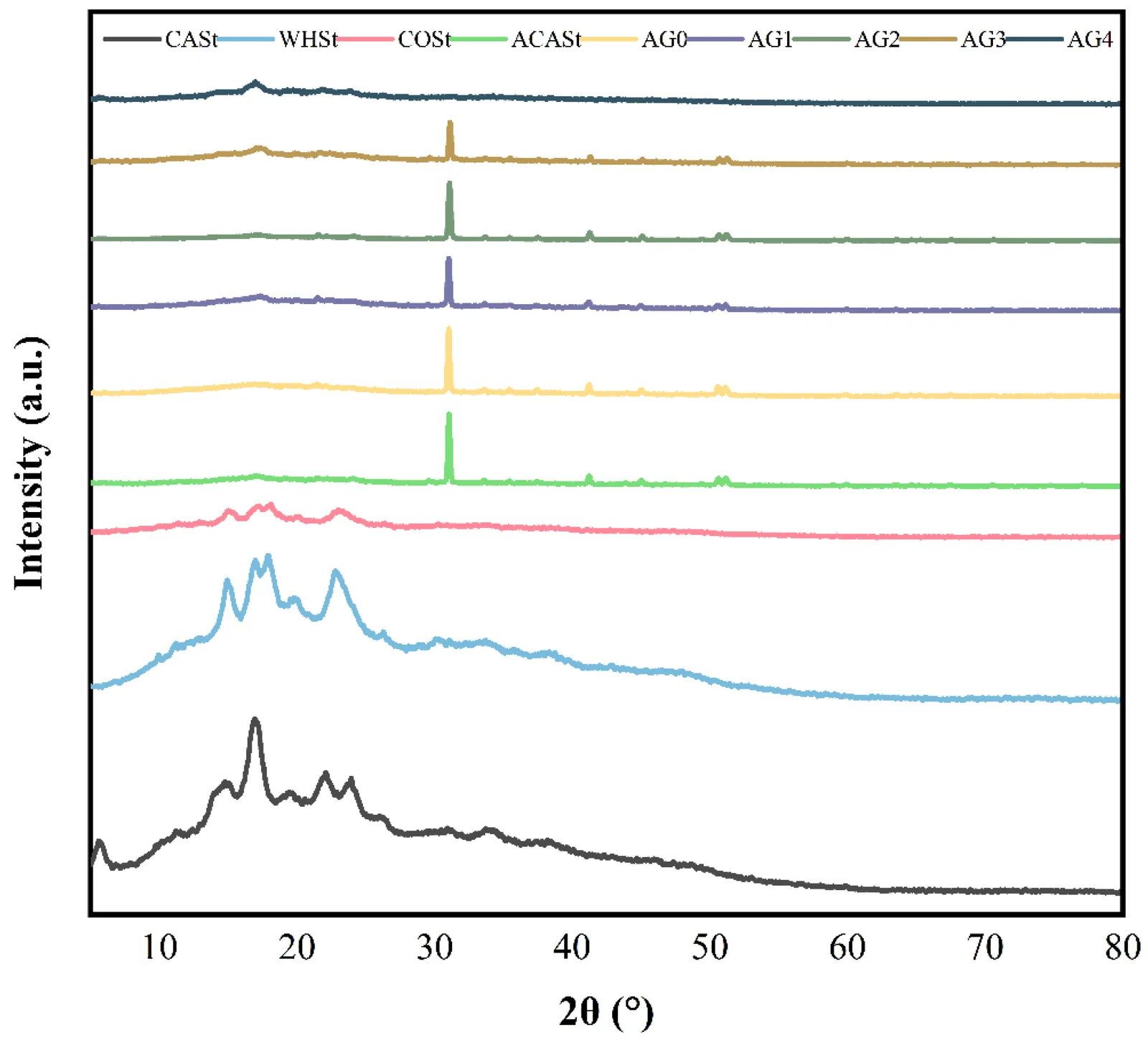
XRD patterns of starch samples and canna starch aerogels.

As shown in Figure 5 and Table 4, the XRD patterns of canna starch (CASt), enzyme-modified starch (ACASt), and the corresponding aerogels indicate changes in crystalline structure after enzymatic treatment and aerogel processing. Native CASt exhibited a typical B-type crystalline pattern, with a main peak at approximately 17° and secondary peaks at 15°, 22°, and 24°. A low-angle peak at around 5.6° was also observed, which is characteristic of B-type crystallinity. Luo et al. reported that this peak is associated with the hydration state, chain packing, and lamellar spacing of amylopectin [32]. The relative crystallinity of CASt was 24.6%, falling within the typical range reported for B-type starches [33]. After α-amylase treatment, ACASt showed a higher apparent relative crystallinity of 89.27% and a crystallite size of 40.55 nm; Kenar et al. [26] reported similar behavior. The increase in apparent relative crystallinity after enzymatic treatment may reflect the preferential hydrolysis of amorphous regions by α-amylase. This selective removal may facilitate the rearrangement and packing of the remaining starch chains into more ordered structures. This increase mainly reflects the enhanced contribution of ordered regions after partial removal of amorphous components rather than the formation of new crystalline structures. The aerogel sample AG0 showed a relative crystallinity of 84.07% with a crystallite size of 40.81 nm, indicating the presence of ordered structures after processing [34]. Compared with native CASt (24.6%), the higher crystallinity of AG0 may be attributed to the structural rearrangement of starch chains during aerogel preparation. Although gelatinization disrupts the original crystalline regions, subsequent cooling and freeze-drying can promote chain reassociation and the formation of ordered structures. This results in higher apparent crystallinity in the aerogel matrix. With increasing ACASt content, the crystallinity varied across samples. AG1 showed 78.99% and 37.20 nm; AG2 showed 92.41% and 36.96 nm; AG3 showed 72.64% and 39.35 nm; and AG4 decreased to 46.89% [35]. Crystallite size remained between 36.96 and 40.81 nm. AG2 having the highest crystallinity suggests that an appropriate ACASt content may facilitate starch chain rearrangement and the formation of ordered regions. However, excessive ACASt addition may disturb the organization of starch chains within the aerogel network, resulting in reduced structural ordering and decreased relative crystallinity in AG3 and AG4. Changes in crystallinity with ACASt addition were also reported in related systems [36].

### 2.6. Thermal Stability Analysis of Starch and Aerogels

**Figure 6:**
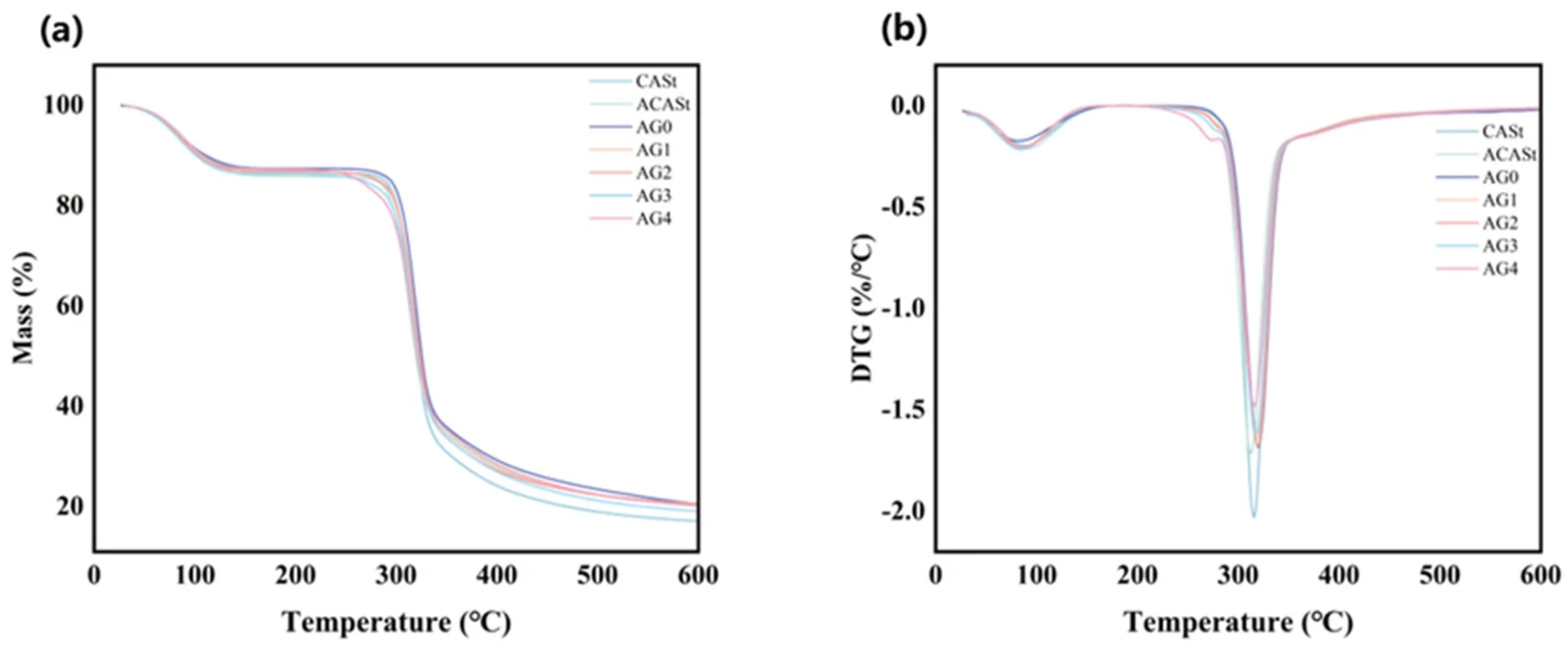
TGA (**a**) and DTG (**b**) curves of starch samples and canna starch aerogels.

The thermal stability and degradation behavior of starch (CASt and ACASt) as well as starch-based aerogels (AG0-−AG4) were evaluated using thermogravimetric analysis (TGA) and differential thermogravimetry (DTG), as illustrated in Figure 6. All samples exhibited three similar weight-loss stages. The initial low-temperature weight loss phase occurring from room temperature (25 °C) to approximately 120 °C was attributed to moisture desorption and loosely bound water. A major mass-loss event between 280 °C and 340 °C followed, corresponding to the primary thermal decomposition of the starch polymer involving glycosidic bond cleavage, dehydration, and depolymerization [37]. A distinct thermal decomposition peak centered at approximately 300 °C suggested that starch was the dominant component responsible for thermal degradation. This is consistent with previous studies on native and modified starches as well as starch-−based aerogels [14,21,38]. A gradual mass loss accompanied by concurrent carbonization was observed when the temperature exceeded 400 °C.

The TGA and DTG curves of CASt, ACASt, and AG0-AG4 were highly similar, which may be attributed to their comparable chemical composition. All samples were mainly composed of starch polysaccharides; thus, their thermal degradation was primarily governed by the decomposition of starch chains, resulting in similar thermal degradation profiles.

Among all samples, the unhydrolyzed AG0 formulation exhibited the highest thermal stability, featuring an onset decomposition temperature (T_onset_) of 303.90 °C and a peak decomposition temperature (T_max_) of 319.64 °C, which were slightly higher than those of native CASt (T_onset_ = 300.53 °C; T_max_ = 315.88 °C). This improvement may be associated with the formation of a relatively stable starch network during gelatinization and subsequent freeze-drying [39].

However, as ACASt substitution increased from 0.5% to 4%, T_onset_ gradually decreased from 301.22 °C to 294.48 °C, whereas T_max_ remained relatively stable (315.88–317.76 °C). This slight variation may reflect partial hydrolysis of starch chains during α-amylase treatment, which altered the molecular arrangement of starch. Meanwhile, moderate chain depolymerization induced by low-dose ACASt may contribute to improved interactions among starch chains and the formation of a more homogeneous gel network. This could partially maintain the thermal stability of AG1 and AG2 [40]. Nevertheless, enzymatic modification did not change the fundamental chemical structure of starch, resulting in similar thermal degradation behaviors among the samples.

Overall, although enzymatic modification and aerogel formation influenced the structural characteristics of starch, their effects on the intrinsic thermal degradation mechanism were limited. Therefore, no pronounced differences were observed in the overall TGA and DTG curves among starch powder, enzyme-modified starch, and starch-based aerogels.

### 2.7. Mechanical and Structural Properties of Aerogels

**Figure 7:**
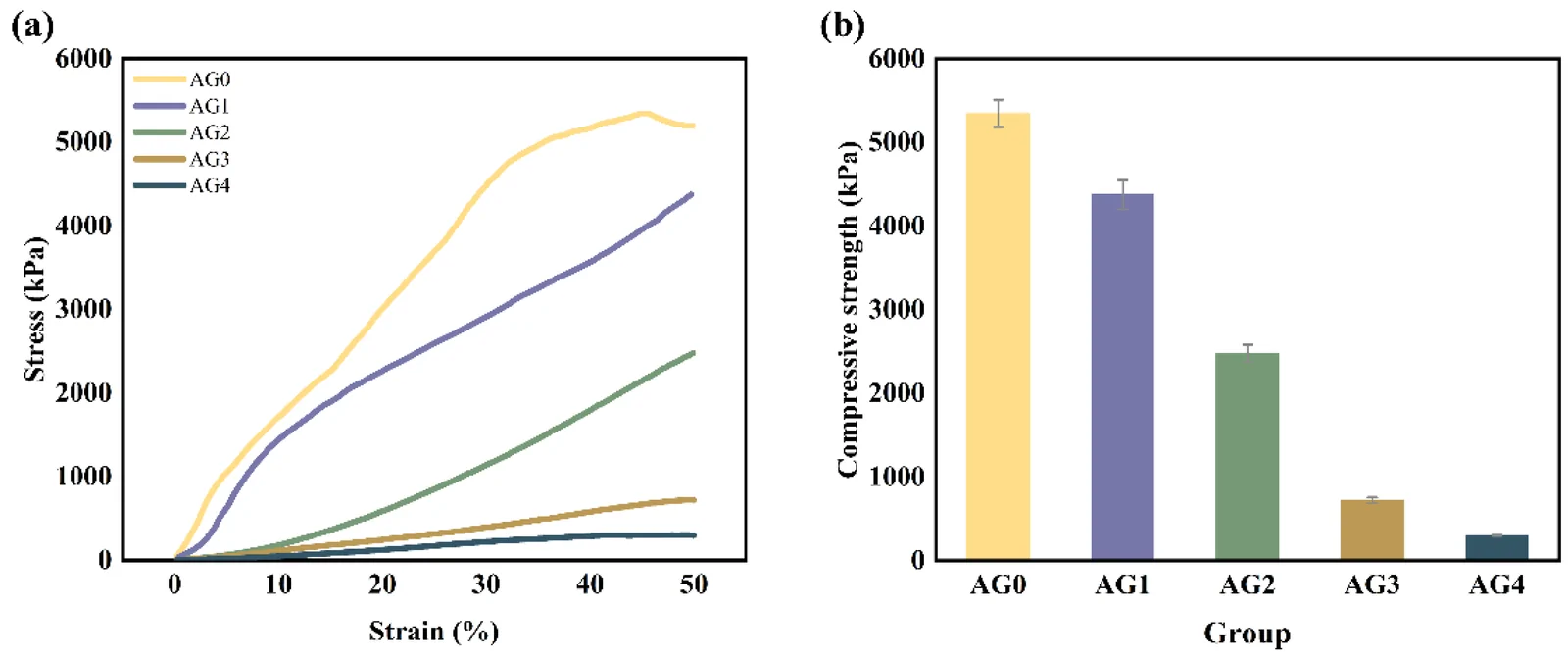
Mechanical properties (**a**) and compressive strength (**b**) of canna starch aerogels.

The mechanical properties of the canna starch aerogels were assessed through stress–strain curves and compressive strength measurements. As illustrated in Figure 7, the aerogels exhibited distinct stress–strain behaviors that vary according to the degree of α-amylase modification, highlighting the interdependence among porosity, network integrity, and load-bearing capacity. The results from compressive strength tests further quantify these effects, providing insights into the trade-offs between mechanical robustness and structural functionality. The stress–strain curves showed that AG0, the unmodified control sample, exhibited the highest load-bearing capacity, with stress values reaching approximately 5000 kPa at 50% strain. This observation suggests that the aerogel maintains a dense and well-interconnected starch network capable of resisting compressive deformation.

The curve for AG0 demonstrates a steep initial rise followed by a gradual increase, characteristic of rigid, mechanically strong [41] porous solids where deformation primarily involves elastic and plastic bending of the network skeleton. Similar findings have been reported for native starch-based aerogels [12], where limited enzymatic or chemical modification leads to a more compact microstructure and enhanced compressive strength. In contrast, AG1 exhibits slightly lower maximum stress (4300–4500 kPa) while maintaining a comparable stress–strain curve to that of AG0. This suggests that limited enzymatic modification resulted in a relatively small reduction in network strength while contributing to increased porosity. Therefore, AG1 exhibited a more favorable balance between structural strength and pore development among the tested aerogels, providing both enhanced structural accessibility and sufficient mechanical stability. The relatively modest decline in compressive strength compared to the control underscores the capacity of partial enzymatic hydrolysis to fine-tune aerogel structures without markedly compromising their load-bearing capabilities. Literature on porous polysaccharide-based aerogels is consistent with this observation [33], suggesting that moderate enzymatic or chemical modification can improve pore connectivity while preserving adequate mechanical integrity.

Further enzymatic treatment, as observed in AG2, AG3, and AG4, leads to marked reductions in compressive performance. Specifically, AG2 exhibits a compressive strength of approximately 2500 kPa, about half that of AG0. This reduction is attributed to increased enzymatic degradation of starch chains, potentially resulting in reduced molecular entanglement and weaker pore-wall cohesion. The stress–strain curve for AG2 no longer displays the steep rise characteristic of AG0 and AG1, indicating lower apparent stiffness and resistance to deformation. For both AG3 and AG4, the compressive strength declines sharply to approximately 700 kPa and below 500 kPa, respectively, signifying an almost complete loss of load-bearing capacity. These highly porous yet mechanically fragile aerogels showed pronounced deformation even under relatively low compressive stress and exhibited limited resistance to further loading. Similar findings have been reported for aerogels derived from over-modified starches and other biopolymers [16], where excessive porosity severely compromises structural cohesion.

The bar chart depicting compressive strength further illustrates these trends by clearly illustrating the descending order of mechanical resistance: AG0 > AG1 > AG2 > AG3 > AG4. From a structure–property perspective, these results underscore the delicate trade-off between porosity and strength. While increased porosity may be advantageous for applications such as adsorption, drug delivery, or insulation, excessive weakening renders the aerogels unsuitable for practical applications requiring mechanical stability [38]. It should be noted that AG1 was not the highest-performing in all individual properties. For example, AG0 exhibited the highest compressive strength, whereas AG2 showed the highest relative crystallinity among the tested samples. Therefore, AG1 was considered the most favorable sample within the tested formulations based on the overall balance between pore accessibility and mechanical stability, rather than as a mathematically optimized formulation. In practical terms, AG1 provided a favorable compromise between higher porosity and mechanical stability, maintaining a compressive strength exceeding 4000 kPa while achieving improved porosity compared with AG0. This combination aligns with documented trends observed in starch- and cellulose-based aerogels [16,42], wherein modest structural modification can yield multifunctional performance.

### 2.8. Equilibrium and Kinetic Study of the Adsorption of C3G with Aerogels

#### 2.8.1. Equilibrium Adsorption Behavior

**Figure 8:**
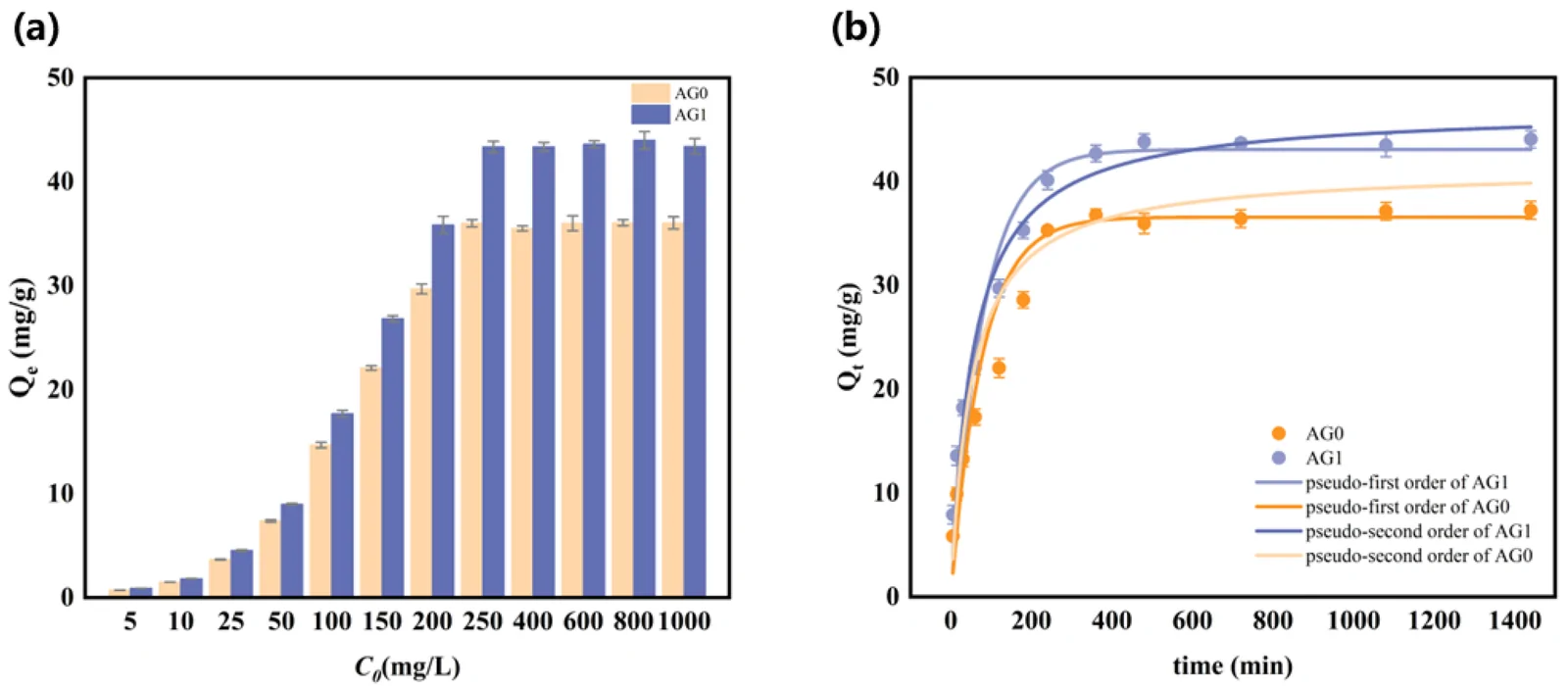
The adsorption performance of AG0 and AG1 for C3G. (**a**) C3G equilibrium adsorption behavior. (**b**) Time curve of C3G adsorption and the fitting curves of the pseudo-first-order kinetic model and the pseudo-second-order kinetic model.

To preliminarily evaluate the influence of α-amylase modification on C3G adsorption behavior, AG0 and AG1 were selected as the unmodified control and the low-dose α-amylase-modified aerogel, respectively. The equilibrium adsorption capacities (*Q_e_*) of AG0 and AG1 at different initial C3G concentrations (*C*_0_) are shown in Figure 8. In general, *Qe* increased with increasing *C*_0_ from 5 to 1000 mg/L. At low concentrations (5–25 mg/L), both aerogels showed relatively low adsorption capacities, which may be attributed to the limited amount of C3G available in solution. Nevertheless, AG1 consistently showed slightly higher *Q_e_* values than AG0 under low-*C*_0_ conditions.

When *C*_0_ increased to 50–200 mg/L, the adsorption capacities of both samples increased rapidly. At 200 mg/L, AG1 reached 35.85 mg/g, while AG0 reached 29.69 mg/g. The higher adsorption capacity of AG1 may be associated with the structural changes induced by α-amylase modification, which could improve the accessibility of adsorption sites. Similar effects of porous structures on adsorption behavior have also been reported in polysaccharide-based composite aerogels containing starch nanoparticles and chitosan nanofibers [43].

At higher concentrations (250–1000 mg/L), the adsorption curves gradually approached a plateau. AG1 showed a saturation adsorption capacity of 43.3–43.9 mg/g, whereas AG0 remained at 35.0–36.0 mg/g. This result is consistent with the gradual saturation of available adsorption sites at high concentrations.

Enzymatic modification may alter the ordered structure of starch and potentially increase the exposure of hydroxyl groups. Since C3G contains multiple hydroxyl groups, hydrogen-bonding interactions may contribute to the adsorption behavior of AG1, although the present results do not directly establish this interaction as the dominant adsorption mechanism. Overall, AG1 exhibited higher adsorption capacity than AG0 over the tested concentration range.

#### 2.8.2. Kinetic Adsorption Behavior

Based on the previously discussed Q_e_-C_0_ relationship, a concentration of 300 mg/L was selected for kinetic analysis. At this concentration, adsorption approached saturation while still showing clear dynamic changes. The kinetic adsorption curves of AG0 and AG1 are shown in Figure 8. Both samples exhibited rapid adsorption during the initial stage (0–120 min), followed by a slower adsorption process. During the initial stage, C3G was quickly adsorbed onto the external surface and onto the readily available adsorption sites. AG1 showed a steeper initial adsorption trend, which may be related to the enhanced accessibility of adsorption sites after α-amylase modification.

After 120 min, the adsorption rate gradually decreased and approached equilibrium. This stage may be associated with the diffusion of C3G into internal pores and its interactions with the starch matrix. Compared with AG0, AG1 maintained a relatively higher adsorption rate during this stage, which may be attributed to the modified starch structure and increased availability of adsorption sites.

As shown in Table 5, the pseudo-second-order model gave higher *R*^2^ values than the pseudo-first-order model for both aerogels. In addition, the calculated *Q_e_* values from the pseudo-second-order model were closer to the experimental results. These results suggest that the pseudo-second-order model was more suitable for describing the adsorption behavior of C3G on the aerogels.

Overall, AG1 exhibited higher adsorption capacity and faster adsorption behavior than AG0, suggesting that α-amylase modification may have increased the accessibility of adsorption sites and enhanced interactions between C3G molecules and the starch-based aerogel matrix, which may have contributed to the observed adsorption behavior.

A limitation of the present study is the absence of Brunauer–Emmett–Teller (BET) (N_2_ adsorption–desorption) analysis, which would provide quantitative data on the specific surface area and micro/mesopore size distribution of the starch aerogels. Given the hierarchical pore structure of these materials, BET pore characterization is warranted and will be the focus of our future work. This will enable robust structure–performance correlations, particularly between nanoscale pore features and the observed adsorption behavior.

## 3. Conclusions

This study successfully developed canna starch aerogels through controlled α-amylase hydrolysis combined with freeze-drying and examined how different hydrolysis levels influenced the aerogel structure and C3G adsorption behavior. Moderate hydrolysis (AG1) partially disrupts the granular architecture and shortens starch chains, which facilitates more uniform ice-crystal templating and yields a highly interconnected porous network (porosity 92%) while preserving network integrity; by contrast, excessive hydrolysis thins the pore walls and weakens inter-chain associations, causing local collapse and a marked loss of compressive strength. This porosity–stability trade-off reflects a general challenge in polysaccharide-based aerogels, where open porosity and mechanical integrity must be balanced through network design.

The pseudo-second-order model provided a better description of the adsorption behavior, while the underlying adsorption mechanism may involve interactions between the hydroxyl-rich starch matrix and the polyphenolic C3G structure, potentially including hydrogen bonding. Compared with recently reported starch-based carriers, the AG1 aerogel achieved a competitive equilibrium adsorption capacity (43.9 mg/g) using only enzymatic restructuring, without chemical crosslinking or nanofiller addition. Overall, controlled enzymatic hydrolysis regulates the structural evolution of canna starch aerogels, and adsorption performance is closely tied to the balance between pore openness and network stability, supporting the potential of enzymatically tailored starch aerogels as sustainable, high-performance carriers for sensitive bioactive compounds in functional food systems.

## 4. Materials and Methods

### 4.1. Materials

Fresh canna tubers were procured from the local market in Wenshan, Yunnan Province, China. Commercial wheat starch and corn starch for control experiments were obtained from Hubei Yucheng E-commerce Co., Ltd. (Jingmen, Hubei, China) and Jingshan Jijiang Food Co., Ltd. ((Jingmen, Hubei, China). Amylopectin standard (87.6%) and amylose standard (from potato) (96.0%) were sourced from Northern Weiye Measurement Group Co., Ltd. (Xinyang, Henan, China). α-Amylase (99%) and cyanidin 3-glucoside (C3G) (98%) were purchased from Aladdin Scientific (Shanghai, China). All other reagents were of analytical grade or above. The water used in the experiment was ultrapure water, prepared in the laboratory.

### 4.2. Extraction of Canna Starch

Fresh canna tubers were cleaned, peeled, and diced. Canna starch was extracted following a previously described method with slight modifications [44]. Briefly, the diced tubers were mixed with distilled water at a solid-to-liquid ratio of 1:4 (*w*/*v*) and homogenized using a commercial blender for 1 min. The homogenate was filtered through eight layers of gauze to remove fibrous residues. After gravitational sedimentation, the supernatant was decanted. The crude starch slurry was resuspended in distilled water at the same 1:4 (*w*/*v*) ratio, thoroughly stirred, and refiltered; this washing cycle was repeated three times. Subsequently, the precipitate was centrifuged at 2000 rpm for 10 min. The supernatant was discarded, and the yellowish impurity layer on the surface of the white starch pellet was manually scraped off. The pellet was resuspended in distilled water and centrifuged again. This purification process was repeated three times until the supernatant became clear. Finally, the purified white starch precipitate was dried in a constant-temperature oven at 40 °C to a constant weight. The dried starch was then pulverized and passed through a 60-mesh sieve to yield a uniform canna starch powder.

### 4.3. Physicochemical Characterization of Canna Starch

#### 4.3.1. Starch Extraction Yield

The starch extraction yield on a dry basis was calculated following the method of Dorantes-Fuertes et al. [45], according to the following equation:

(1) $$\mathrm{Ed}\left(\%\right)=\frac{W}{M-(M\times U)}\times 100$$

where Ed is the extraction yield on a dry basis (%), *W* is the extracted starch mass (g), *M* is the raw material mass (g), and *U* is the moisture content of the raw material (%).

#### 4.3.2. Characterization of the Composition of Canna Starch

The moisture, ash, protein, and fat contents of the starches were determined following the procedures outlined by Su et al. [46]. The amylose and amylopectin contents were quantified based on the method of Lucas-Aguirre et al. with slight modifications [47].

#### 4.3.3. Water Absorption Index, Water Solubility Index, and Swelling Power of Starch

The water absorption index (WAI), water solubility index (WSI), and swelling power (SP) of the canna starch were evaluated following the protocol described by Martínez-Rubio et al. [48]. Commercial wheat and corn starches were used as references for comparative analysis.

Briefly, an accurately weighed starch sample (0.5 g, dry basis) was dispersed in 30 mL of distilled water in a 100 mL Erlenmeyer flask. The mixture was continuously agitated to form a uniform suspension, placed in a thermostatic oil bath with magnetic stirring (500 rpm), and heated at target temperatures (65 °C, 75 °C, 85 °C, or 95 °C) for 30 min. After gelatinization, the suspension was cooled to room temperature and centrifuged at 5000 rpm for 15 min, separating the mixture into a clear supernatant and a wet precipitate.

The entire supernatant was transferred into a pre-weighed Petri dish and dried in an oven at 105 °C to a constant weight to determine the soluble residue mass. Concurrently, the centrifuge tube containing the wet precipitate was inverted on absorbent paper for 10 min to drain excess free water, and the wet precipitate was weighed. Finally, the precipitate was dried at 105 °C to a constant weight to record its dry mass. WAI, WSI, and SP were calculated according to the Equations (2)–(4):

(2) $$\mathrm{WAI}=\frac{\mathrm{PRC}}{\mathrm{PA}-\mathrm{PRE}}$$

(3) $$\mathrm{WSI}=\frac{\mathrm{PRE}}{\mathrm{PA}}\times 100$$

(4) $$\mathrm{SP}=\frac{\mathrm{PRC}-\mathrm{PRCD}}{\mathrm{PA}}\times 100$$

where *PRC* is the weight of wet centrifugal precipitation (g), *PRCD* is the dry weight of the precipitate (g), *PA* is the initial dry weight of the starch sample (g), and *PRE* is the weight of the dried supernatant residue (g).

### 4.4. Enzymatic Modification of Canna Starch

Enzymatically modified canna starch was prepared based on the protocol of Li et al. [49] with minor modifications. Briefly, an aqueous starch suspension (1:6, *w*/*w*) in an acetic acid-sodium acetate buffer (pH 4.8) was incubated in a 50 °C oil bath under continuous magnetic stirring (200 rpm). α-Amylase (activity ≥ 500 KNU/g) was introduced at 1.5% (*w*/*w*, dry starch basis), and the hydrolysis was carried out at room temperature for 8 h until the dextrose equivalent (DE) reached 4 ± 0.5.

The reaction was rapidly terminated by neutralizing the mixture to pH 7.0 with 1 M NaOH. Following centrifugation (8000 rpm, 10 min), the obtained starch pellet was washed in triplicate with distilled water, pre-frozen at −80 °C, and lyophilized for 48 h. The resultant powder was sifted through a 250-mesh sieve to obtain the modified starch, denoted as ACASt.

### 4.5. Preparation of Starch-Based Aerogels

Aqueous suspensions with a total solid concentration of 10% (*w*/*v*) were prepared by blending ACASt and unmodified canna starch using distilled water as the solvent. The mass ratios of ACASt to unmodified starch were set at 0:10, 0.5:9.5, 1:9, 2.5:7.5, and 4:6. The suspensions were gelatinized in an oil bath at 105 °C for 15 min under continuous magnetic stirring at 500 rpm. Immediately after gelatinization, the hot starch paste was cast into 6-well cylindrical molds (internal diameter 3.5 cm, height 2.3 cm). The samples were subjected to a stepwise cooling program (25 °C, followed by 4 °C, and finally pre-frozen at −80 °C). Once fully frozen, the samples were lyophilized (−80 °C, 48 h) to yield the composite aerogels. The resulting aerogels were designated as AG0, AG1, AG2, AG3, and AG4, corresponding to their respective mass ratios, with AG0 serving as the blank control.

### 4.6. Physical Properties of Starch-Based Aerogels

#### 4.6.1. Mechanical Properties

The mechanical properties of the composite aerogels were evaluated using a texture analyzer (Brookfield CT3, Middleboro, MA, USA). Uniaxial compression tests were performed to determine the compressive strength and obtain the stress–strain curves of the samples. During the testing, each aerogel sample was compressed by a 5-mm diameter cylindrical probe at a constant crosshead speed of 0.2 mm/s.

#### 4.6.2. Density and Porosity

The density of the aerogels was determined using a fine sand displacement method [50]. Briefly, a graduated cylinder was partially filled with fine sand and leveled. The aerogel sample was then placed into the cylinder and covered with additional fine sand to completely fill any void spaces between the sample and the cylinder walls. This initial total volume (*V*1) was recorded. After carefully removing the aerogel, the remaining sand was leveled again, and the second volume (*V*2) was recorded. The volume (*V*) and apparent density (*ρ*) of the aerogels were calculated according to the following equation:

(5) V=V2−V1

(6) $$\rho =\frac{V}{M}$$

where *V*1 is the initial volume reading of the graduated cylinder, *V*2 is the second volume reading after aerogel removal, and *M* is the mass of the dry aerogel.

The porosity was determined based on the liquid displacement method described by Horvat et al. [51], with minor modifications. The pre-weighed dry aerogel was immersed in ethanol until saturation. Upon removal, excess ethanol on the surface was gently wiped off using filter paper, and the wet sample was immediately reweighed. The porosity of the aerogels was calculated according to the following equation:

(7) $$\mathrm{Porosity}\left(\%\right)=\frac{M2-M1}{\rho V}\times 100$$

where *M*1 is the mass of the dry aerogel (g), *M*2 is the mass of the wet aerogel after ethanol immersion (g), *V* is the volume of the dry aerogel obtained from Equation (7) (cm^3^), and *ρ* is the density of ethanol (0.785 g cm^−3^).

### 4.7. Scanning Electron Microscope (SEM)

The morphology and size of the starch granules, as well as the porous structure of the aerogels, were evaluated using SEM (S-3000N, Hitachi, Tokyo, Japan) based on the method of Xiang et al. [52]. Starch powder samples were dusted onto double-sided conductive carbon tape, while the aerogel samples were sectioned and similarly affixed. All samples were sputter-coated with gold prior to observation. The micrographs were acquired at an accelerating voltage of 15 kV, with magnifications of 250× and 500× for both starch granules and aerogel sections. Following the procedure outlined by Boruczkowski et al. [53], the sizes of at least 30 individual starch granules per sample were measured from the SEM images using ImageJ software (version 1.54r).

### 4.8. Fourier Transform Infrared (FT-IR)

Various starches (unmodified canna, wheat, and corn starches), ACASt, and the composite aerogels were prepared for FT-IR analysis. The spectra were recorded using a Nicolet 5700 FT-IR spectrometer (Thermo Nicolet, Madison, WI, USA) in transmittance mode at room temperature. Each spectrum was acquired over a wavenumber range of 4000 to 400 cm^−1^, accumulating 32 scans with a spectral resolution of 4 cm^−1^, according to the methodology described by Warren et al. [30]. The transmittance spectra were converted to absorbance using A = −lgT, where T represents transmittance expressed as a decimal fraction. The absorbance values at approximately 1047, 1022, and 995 cm^−1^ in the fingerprint region (1200–800 cm^−1^) were obtained from the converted spectra, and the absorbance ratios R_1047/1022_ (ratio of absorbance at 1047 cm^−1^ to that at 1022 cm^−1^) and R_1022/995_ (ratio of absorbance at 1022 cm^−1^ to that at 995 cm^−1^) were calculated and used as semi-quantitative indicators of short-range molecular order and changes in the hydrogen-bonding environment, respectively.

### 4.9. X-Ray Diffraction (XRD)

The crystalline structures of the starch and aerogel samples were characterized using an X-ray diffractometer (D8 Advance, Bruker, Karlsruhe, Germany) [54]. The diffractograms were recorded over a 2θ scanning range of 5° to 80°. The instrument was operated at a voltage of 40 kV and a current of 40 mA, with a scanning speed of 1°/min.

Calculations for relative crystallinity (RC) and crystallite size were based on the method reported by Barros et al. [9]. The XRD patterns were first baseline-corrected, and the crystalline peaks were separated from the amorphous region by peak deconvolution using Origin software (version 2024). The RC was calculated based on the ratio of the integrated crystalline peak area to the total fitted area, following the approach of Staker et al. [55]. When severe overlap occurred between crystalline and amorphous regions, peak deconvolution was applied to obtain reliable separation, and crystallite size calculations were correspondingly excluded. The RC was determined according to the following equation:

(8) $$\mathrm{RC}=\frac{A_{c}}{A_{c}+A_{a}}\times 100\%$$

where $A_{c}$ is the integrated area of the crystalline peaks and $A_{a}$ is the integrated area of the amorphous region.

The crystallite size (D) was estimated according to the following equation:

(9) $$D=\frac{K\lambda }{\beta \cos\theta }$$

where *K* is a constant, λ is the X-ray wavelength, β is the full width at half maximum of the peak, and θ is the Bragg angle.

### 4.10. Thermal Stability Analysis

The thermal stability of the samples was evaluated via thermogravimetric analysis (TGA) using a TA Q500 thermal analyzer (TA Instruments, New Castle, DE, USA) under a dynamic nitrogen atmosphere. Based on the method of Staker et al. with minor modifications [55], approximately 5.0 mg of each sample was placed in a crucible and heated from 30 °C to 600 °C at a constant heating rate of 10 °C∙min^−1^.

### 4.11. Adsorption Performance of ACASt Aerogels for C3G

#### 4.11.1. Equilibrium Adsorption of C3G

Equilibrium C3G adsorption experiments were conducted according to the method described by Fan et al. [56], with minor modifications. The ACASt aerogels (AG0 and AG1) were ground and passed through a 50-mesh sieve. Subsequently, 50 mg of the aerogel powder was added to 10 mL amber glass bottles containing 10 mL of aqueous C3G solutions with varying initial concentrations (C_0_ = 5–1000 mg/L). The sealed bottles were incubated in a thermostatic shaker at 150 rpm and 25 °C for 24 h to reach adsorption equilibrium. Following incubation, the suspensions were filtered through a 0.22 μm membrane filter, and the equilibrium concentrations (*C_e_*) of C3G in the filtrates were determined spectrophotometrically at 520 nm. The equilibrium adsorption capacity, Q_e_ (mg/g), was calculated according to the following equation:

(10) $$Q_{e}=\frac{(C_{0}-C_{e})\times V\times N}{M}$$

where *C*_0_ is the initial concentration of C3G in the solution (mg/L), *C_e_* is the equilibrium concentration of C3G in the solution (mg/L), *V* is the volume of the C3G solution (mL), *M* is the mass of the adsorbent (mg), and *N* is the dilution factor.

#### 4.11.2. Adsorption Kinetics of C3G

The adsorption kinetics of C3G were investigated using a modified procedure. To ensure the kinetic profile captured both the rapid initial uptake and the subsequent intraparticle diffusion stage, the initial C3G concentration was selected based on the isothermal equilibrium results. A concentration near adsorption saturation was targeted. Briefly, 50 mg of the sieved aerogel powder (50-mesh) was dispersed in 10 mL of the selected C3G solution in amber bottles and agitated at 150 rpm and 25 °C. At predetermined time intervals (5–1440 min), aliquots of the suspension were withdrawn, filtered (0.22 μm), and analyzed at 520 nm. The adsorption capacity at time (Q_t_) (mg/g), was calculated according to the following equation:

(11) $$Q_{t}=\frac{(C_{0}-C_{t})\times V\times N}{M}$$

where *C_t_* is the concentration of C3G in the liquid phase at time t (mg/L). The variables *C*_0_, *V*, *M*, and *N* are defined identically to those in Equation (10). Both pseudo-first-order and pseudo-second-order kinetic models were employed to fit the experimental data.

### 4.12. Statistical Analysis

All experiments were performed in triplicate, and the results are expressed as the mean ± standard deviation (SD). Data regarding the physicochemical parameters of the starch samples, as well as the density and porosity of the aerogels, were evaluated using a one-way analysis of variance (ANOVA). Differences between the mean values were determined using Tukey’s test, and statistical significance was defined at a 95% confidence level (*p* < 0.05).

## Figures and Tables

**Table 1:** Physicochemical characteristics of *Canna edulis*, wheat, and corn starch samples.

Parameter	Canna Starch	Wheat Starch	Corn Starch
Ash (%)	0.31 ± 0.02 ^b^	0.42 ± 0.03 ^c^	0.25 ± 0.01 ^a^
Crude Protein (%)	0.70 ± 0.05 ^b^	0.28 ± 0.02 ^a^	0.30 ± 0.01 ^a^
Crude fat (%)	0.05 ± 0.005 ^a^	0.10 ± 0.01 ^c^	0.07 ± 0.008 ^b^
Amylose (%)	18.35 ± 0.5 ^a^	21.18 ± 0.6 ^b^	24.53 ± 0.7 ^c^
Amylopectin (%)	80.63 ± 1.0 ^c^	78.09 ± 0.8 ^b^	74.88 ± 0.9 ^a^
Moisture (%)	13.36 ± 0.4 ^c^	11.40 ± 0.3 ^a^	12.02 ± 0.2 ^b^
Total starch (%)	98.99 ± 0.3 ^a^	99.27 ± 0.2 ^b^	99.41 ± 0.1 ^b^

Values are mean ± SD (*n* = 3). ^a,b,c^: Values with different lowercase letters among the canna starch, wheat starch, and corn starch groups show a significant difference in a Tukey test (*p* < 0.05).

**Table 2:** Absorbance ratio values of starch samples and aerogels AG0-AG4.

Sample	R_1047/1022_	R_1022/995_
CASt	0.9712	1.0120
WHSt	1.0541	1.0417
COSt	0.9773	0.9851
ACASt	0.8545	0.7322
AG0	0.9773	0.7230
AG1	1.3718	1.0000
AG2	1.3863	1.0003
AG3	0.9988	0.9882
AG4	0.9990	0.9851

**Table 3:** Density and porosity of aerogels AG0–AG4.

Group	Density (g/cm^3^)	Porosity (%)
AG0	0.11 ± 0.0029 ^c^	86 ± 3.67 ^a^
AG1	0.10 ± 0.0031 ^b^	92 ± 2.82 ^c^
AG2	0.09 ± 0.0029 ^a^	92 ± 2.51 ^c^
AG3	0.09 ± 0.0051 ^a^	91 ± 4.29 ^b^
AG4	0.09 ± 0.0035 ^a^	91 ± 1.94 ^b^

Values are mean ± SD (*n* = 3). ^a,b,c^: Values with different lowercase letters in the same column show a significant difference in a Tukey test (*p* < 0.05).

**Table 4:** Relative crystallinity and grain size of enzyme-modified starch-based aerogels.

Sample	Relative Crystallinity (%)	Crystallite Size (nm)
CASt	24.60	-
ACASt	89.27	40.55
AG0	84.07	40.81
AG1	78.99	37.20
AG2	92.41	36.96
AG3	72.64	39.35
AG4	46.89	-

**Table 5:** Kinetic parameters of C3G adsorption by aerogel.

Kinetic Model	Parameter	AG0	AG1
Pseudo-first-order	k_1_ (g/mg min)	0.0129	0.01255
	Q_e_ (mg/g)	36.56181	43.07036
	R^2^	0.96326	0.94272
Pseudo-second-order	k_2_ (g/mg min)	0.00047	0.00039
	Q_e_ (mg/g)	41.23448	46.93357
	R^2^	0.96799	0.97203

Values are mean ± SD (*n* = 3). Differences between groups were considered statistically significant (*p* < 0.05).

## Data Availability

The original contributions presented in this study are included in the article. Further inquiries can be directed to the corresponding author.
